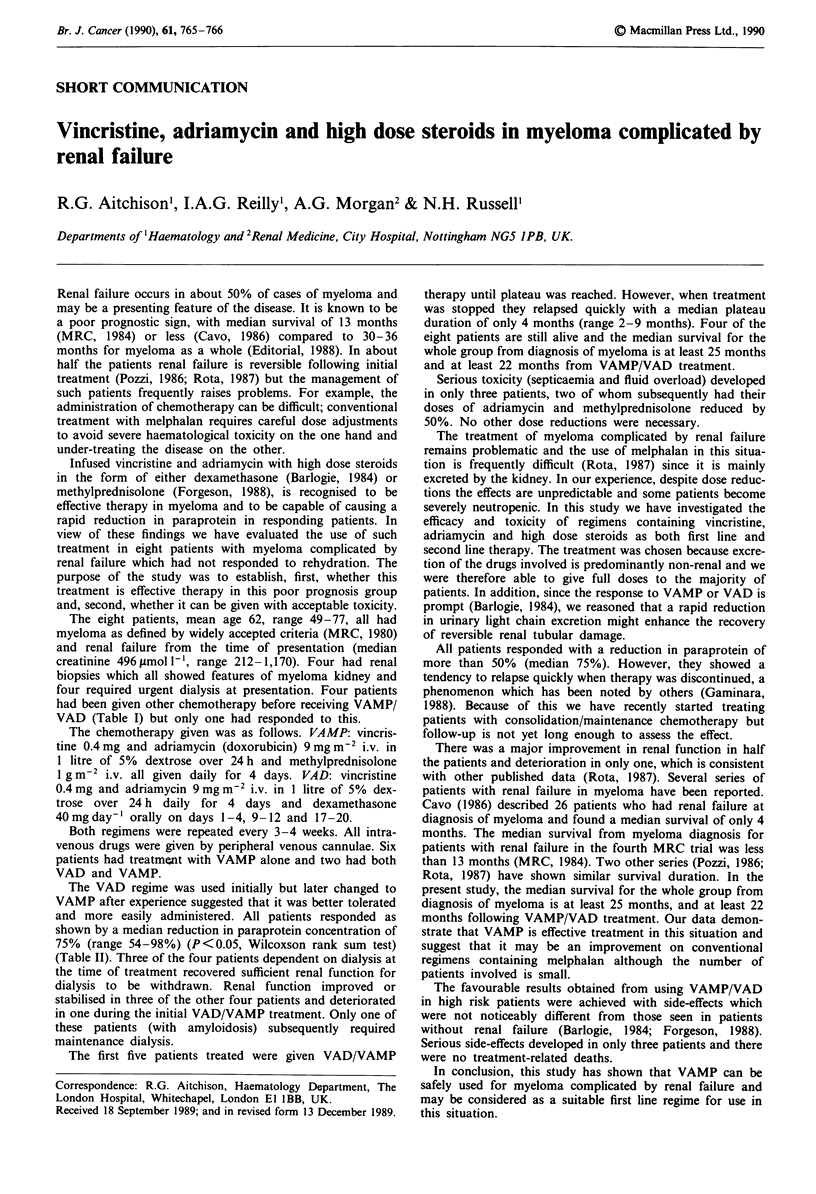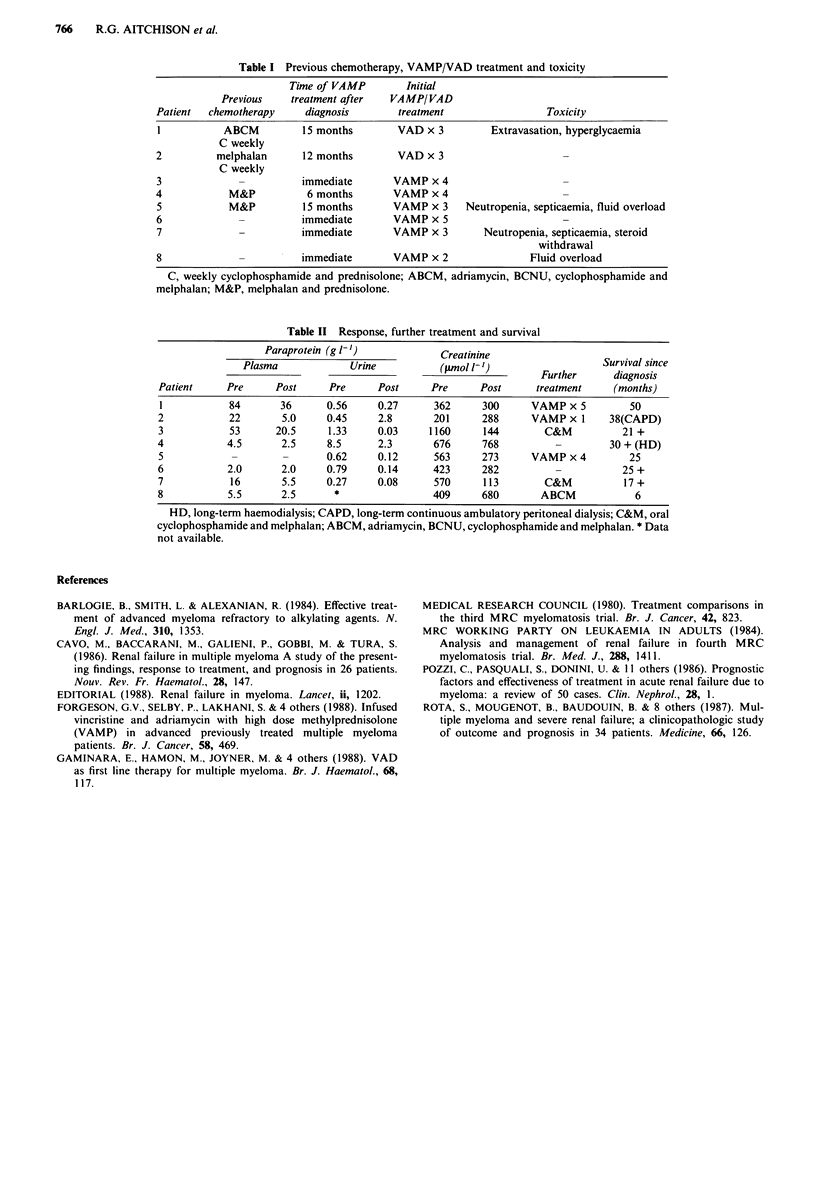# Vincristine, adriamycin and high dose steroids in myeloma complicated by renal failure.

**DOI:** 10.1038/bjc.1990.171

**Published:** 1990-05

**Authors:** R. G. Aitchison, I. A. Reilly, A. G. Morgan, N. H. Russell

**Affiliations:** Department of Haematology, City Hospital, Nottingham, UK.


Br. J. Cancer (1990), 61, 765-766 © Macmillan Press Ltd., 1990

SHORT COMMUNICATION

### Vincristine, adriamycin and high dose steroids in myeloma complicated by renal failure

R.G. Aitchison[1], I.A.G. Reilly[1], A.G. Morgan[2] & N.H. Russell[1]

*Departments of [1]Haematology and [2]Renal Medicine, City Hospital, Nottingham NG5 1PB, UK.*

Renal failure occurs in about 50% of cases of myeloma and may be a presenting feature of the disease. It is known to be a poor prognostic sign, with median survival of 13 months (MRC, 1984) or less (Cavo, 1986) compared to 30-36 months for myeloma as a whole (Editorial, 1988). In about half the patients renal failure is reversible following initial treatment (Pozzi, 1986; Rota, 1987) but the management of such patients frequently raises problems. For example, the administration of chemotherapy can be difficult; conventional treatment with melphalan requires careful dose adjustments to avoid severe haematological toxicity on the one hand and under-treating the disease on the other.

Infused vincristine and adriamycin with high dose steroids in the form of either dexamethasone (Barlogie, 1984) or methylprednisolone (Forgeson, 1988), is recognised to be effective therapy in myeloma and to be capable of causing a rapid reduction in paraprotein in responding patients. In view of these findings we have evaluated the use of such treatment in eight patients with myeloma complicated by renal failure which had not responded to rehydration. The purpose of the study was to establish, first, whether this treatment is effective therapy in this poor prognosis group and, second, whether it can be given with acceptable toxicity.

The eight patients, mean age 62, range 49-77, all had myeloma as defined by widely accepted criteria (MRC, 1980) and renal failure from the time of presentation (median creatinine 496 $\mu mol\ l^{-1}$, range 212-1,170). Four had renal biopsies which all showed features of myeloma kidney and four required urgent dialysis at presentation. Four patients had been given other chemotherapy before receiving VAMP/VAD (Table I) but only one had responded to this.

The chemotherapy given was as follows. *VAMP*: vincristine 0.4 mg and adriamycin (doxorubicin) 9 mg $m^{-2}$ i.v. in 1 litre of 5% dextrose over 24 h and methylprednisolone 1 g $m^{-2}$ i.v. all given daily for 4 days. *VAD*: vincristine 0.4 mg and adriamycin 9 mg $m^{-2}$ i.v. in 1 litre of 5% dextrose over 24 h daily for 4 days and dexamethasone 40 mg $day^{-1}$ orally on days 1-4, 9-12 and 17-20.

Both regimens were repeated every 3-4 weeks. All intravenous drugs were given by peripheral venous cannulae. Six patients had treatment with VAMP alone and two had both VAD and VAMP.

The VAD regime was used initially but later changed to VAMP after experience suggested that it was better tolerated and more easily administered. All patients responded as shown by a median reduction in paraprotein concentration of 75% (range 54-98%) ($P < 0.05$, Wilcoxson rank sum test) (Table II). Three of the four patients dependent on dialysis at the time of treatment recovered sufficient renal function for dialysis to be withdrawn. Renal function improved or stabilised in three of the other four patients and deteriorated in one during the initial VAD/VAMP treatment. Only one of these patients (with amyloidosis) subsequently required maintenance dialysis.

The first five patients treated were given VAD/VAMP therapy until plateau was reached. However, when treatment was stopped they relapsed quickly with a median plateau duration of only 4 months (range 2-9 months). Four of the eight patients are still alive and the median survival for the whole group from diagnosis of myeloma is at least 25 months and at least 22 months from VAMP/VAD treatment.

Serious toxicity (septicaemia and fluid overload) developed in only three patients, two of whom subsequently had their doses of adriamycin and methylprednisolone reduced by 50%. No other dose reductions were necessary.

The treatment of myeloma complicated by renal failure remains problematic and the use of melphalan in this situation is frequently difficult (Rota, 1987) since it is mainly excreted by the kidney. In our experience, despite dose reductions the effects are unpredictable and some patients become severely neutropenic. In this study we have investigated the efficacy and toxicity of regimens containing vincristine, adriamycin and high dose steroids as both first line and second line therapy. The treatment was chosen because excretion of the drugs involved is predominantly non-renal and we were therefore able to give full doses to the majority of patients. In addition, since the response to VAMP or VAD is prompt (Barlogie, 1984), we reasoned that a rapid reduction in urinary light chain excretion might enhance the recovery of reversible renal tubular damage.

All patients responded with a reduction in paraprotein of more than 50% (median 75%). However, they showed a tendency to relapse quickly when therapy was discontinued, a phenomenon which has been noted by others (Gaminara, 1988). Because of this we have recently started treating patients with consolidation/maintenance chemotherapy but follow-up is not yet long enough to assess the effect.

There was a major improvement in renal function in half the patients and deterioration in only one, which is consistent with other published data (Rota, 1987). Several series of patients with renal failure in myeloma have been reported. Cavo (1986) described 26 patients who had renal failure at diagnosis of myeloma and found a median survival of only 4 months. The median survival from myeloma diagnosis for patients with renal failure in the fourth MRC trial was less than 13 months (MRC, 1984). Two other series (Pozzi, 1986; Rota, 1987) have shown similar survival duration. In the present study, the median survival for the whole group from diagnosis of myeloma is at least 25 months, and at least 22 months following VAMP/VAD treatment. Our data demonstrate that VAMP is effective treatment in this situation and suggest that it may be an improvement on conventional regimens containing melphalan although the number of patients involved is small.

The favourable results obtained from using VAMP/VAD in high risk patients were achieved with side-effects which were not noticeably different from those seen in patients without renal failure (Barlogie, 1984; Forgeson, 1988). Serious side-effects developed in only three patients and there were no treatment-related deaths.

In conclusion, this study has shown that VAMP can be safely used for myeloma complicated by renal failure and may be considered as a suitable first line regime for use in this situation.

Correspondence: R.G. Aitchison, Haematology Department, The London Hospital, Whitechapel, London E1 1BB, UK.
Received 18 September 1989; and in revised form 13 December 1989.

**Table I** Previous chemotherapy, VAMP/VAD treatment and toxicity

| *Patient* | *Previous chemotherapy* | *Time of VAMP treatment after diagnosis* | *Initial VAMP/VAD treatment* | *Toxicity* |
|---|---|---|---|---|
| 1 | ABCM | 15 months | VAD x 3 | Extravasation, hyperglycaemia |
| 2 | C weekly melphalan C weekly | 12 months | VAD x 3 | – |
| 3 | – | immediate | VAMP x 4 | – |
| 4 | M&P | 6 months | VAMP x 4 | – |
| 5 | M&P | 15 months | VAMP x 3 | Neutropenia, septicaemia, fluid overload |
| 6 | – | immediate | VAMP x 5 | – |
| 7 | – | immediate | VAMP x 3 | Neutropenia, septicaemia, steroid withdrawal |
| 8 | – | immediate | VAMP x 2 | Fluid overload |

C, weekly cyclophosphamide and prednisolone; ABCM, adriamycin, BCNU, cyclophosphamide and melphalan; M&P, melphalan and prednisolone.

**Table II** Response, further treatment and survival

| | *Paraprotein ($g\ l^{-1}$)* | | | | *Creatinine ($\mu mol\ l^{-1}$)* | | | |
|---|---|---|---|---|---|---|---|---|
| | *Plasma* | | *Urine* | | | | | |
| *Patient* | *Pre* | *Post* | *Pre* | *Post* | *Pre* | *Post* | *Further treatment* | *Survival since diagnosis (months)* |
| 1 | 84 | 36 | 0.56 | 0.27 | 362 | 300 | VAMP x 5 | 50 |
| 2 | 22 | 5.0 | 0.45 | 2.8 | 201 | 288 | VAMP x 1 | 38(CAPD) |
| 3 | 53 | 20.5 | 1.33 | 0.03 | 1160 | 144 | C&M | 21 + |
| 4 | 4.5 | 2.5 | 8.5 | 2.3 | 676 | 768 | – | 30 + (HD) |
| 5 | – | – | 0.62 | 0.12 | 563 | 273 | VAMP x 4 | 25 |
| 6 | 2.0 | 2.0 | 0.79 | 0.14 | 423 | 282 | – | 25 + |
| 7 | 16 | 5.5 | 0.27 | 0.08 | 570 | 113 | C&M | 17 + |
| 8 | 5.5 | 2.5 | * | | 409 | 680 | ABCM | 6 |

HD, long-term haemodialysis; CAPD, long-term continuous ambulatory peritoneal dialysis; C&M, oral cyclophosphamide and melphalan; ABCM, adriamycin, BCNU, cyclophosphamide and melphalan. * Data not available.

### References

BARLOGIE, B., SMITH, L. & ALEXANIAN, R. (1984). Effective treatment of advanced myeloma refractory to alkylating agents. *N. Engl. J. Med.*, **310**, 1353.

CAVO, M., BACCARANI, M., GALIENI, P., GOBBI, M. & TURA, S. (1986). Renal failure in multiple myeloma A study of the presenting findings, response to treatment, and prognosis in 26 patients. *Nouv. Rev. Fr. Haematol.*, **28**, 147.

EDITORIAL (1988). Renal failure in myeloma. *Lancet*, **ii**, 1202.

FORGESON, G.V., SELBY, P., LAKHANI, S. & 4 others (1988). Infused vincristine and adriamycin with high dose methylprednisolone (VAMP) in advanced previously treated multiple myeloma patients. *Br. J. Cancer*, **58**, 469.

GAMINARA, E., HAMON, M., JOYNER, M. & 4 others (1988). VAD as first line therapy for multiple myeloma. *Br. J. Haematol.*, **68**, 117.

MEDICAL RESEARCH COUNCIL (1980). Treatment comparisons in the third MRC myelomatosis trial. *Br. J. Cancer*, **42**, 823.

MRC WORKING PARTY ON LEUKAEMIA IN ADULTS (1984). Analysis and management of renal failure in fourth MRC myelomatosis trial. *Br. Med. J.*, **288**, 1411.

POZZI, C., PASQUALI, S., DONINI, U. & 11 others (1986). Prognostic factors and effectiveness of treatment in acute renal failure due to myeloma: a review of 50 cases. *Clin. Nephrol.*, **28**, 1.

ROTA, S., MOUGENOT, B., BAUDOUIN, B. & 8 others (1987). Multiple myeloma and severe renal failure; a clinicopathologic study of outcome and prognosis in 34 patients. *Medicine*, **66**, 126.